# Non-Thermal Plasma (NTP) Treatment of Alfalfa Seeds in Different Voltage Conditions Leads to Both Positive and Inhibitory Outcomes Related to Sprout Growth and Nutraceutical Properties

**DOI:** 10.3390/plants13081140

**Published:** 2024-04-19

**Authors:** Iuliana Motrescu, Constantin Lungoci, Anca Elena Calistru, Camelia Elena Luchian, Tincuta Marta Gocan, Cristina Mihaela Rimbu, Emilian Bulgariu, Mihai Alexandru Ciolan, Gerard Jitareanu

**Affiliations:** 1 Department of Exact Sciences, “Ion Ionescu de la Brad” Iasi University of Life Sciences, 700490 Iasi, Romania; camelia.luchian@iuls.ro (C.E.L.); emilian.bulgariu@iuls.ro (E.B.); 2 Research Institute for Agriculture and Environment, “Ion Ionescu de la Brad” Iasi University of Life Sciences, 700490 Iasi, Romania; anca.calistru@iuls.ro (A.E.C.); gerard.jitareanu@iuls.ro (G.J.); 3 Department of Plant Sciences, “Ion Ionescu de la Brad” Iasi University of Life Sciences, 700490 Iasi, Romania; constantin.lungoci@iuls.ro; 4 Department of Pedotechnics, “Ion Ionescu de la Brad” Iasi University of Life Sciences, 700490 Iasi, Romania; 5 Faculty of Horticulture and Business in Rural Development, University of Agricultural Sciences and Veterinary Medicine Cluj-Napoca, 400372 Cluj-Napoca, Romania; tincuta.gocan@usamvcluj.ro; 6 Department of Public Health, “Ion Ionescu de la Brad” Iasi University of Life Sciences, 700490 Iasi, Romania; cristina.rimbu@iuls.ro; 7 Research Center on Advanced Materials and Technologies, Department of Exact and Natural Science, Institute of Interdisciplinary Research, Alexandru Ioan Cuza University of Iasi, 700506 Iasi, Romania; mihai.ciolan@uaic.ro

**Keywords:** non-thermal plasma, plasma treatment, sprouts, nutraceuticals, alfalfa

## Abstract

Non-thermal plasma (NTP) has proven to be a green method in the agricultural field for the stimulation of germination, growth, and production of nutraceutical compounds in some cases. However, the process is far from being fully understood and depends on the targeted plant species and the NTP used. In this work, we focus on the production of alfalfa sprouts from NTP-treated seeds under different voltage conditions. A flexible electrode configuration was used to produce the NTP, which can also be placed on packages for in-package treatments. The surface of the seeds was analyzed, indicating that the microstructure was strongly affected by NTP treatment. Biometric measurements evidenced the possibility of stimulating the sprout growth in some conditions by up to 50% compared to the sprouts obtained from untreated seeds. Biochemical traits for the sprouts obtained in different processing conditions were also studied, such as the concentrations of chlorophyll pigments, flavonoids and polyphenols, and antioxidant activity. Most NTP treatments led to inhibitory effects, proving the strong dependence between NTP treatment and targeted plant species.

## 1. Introduction

The field of plasma agriculture has been rapidly growing due to the many advantages of non-thermal treatments in applications such as scarification, stimulation of seed germination, growth, and enhancing the nutraceutical properties of the plants resulting from seed treatments, all accompanied by the inactivation of the microbiological load that might be present on the surface of the seeds [1,2,3,4,5,6,7,8,9,10]. Applications are numerous and intensively studied, non-thermal plasma processing for agricultural purposes being a technology that does not involve the use of any dangerous chemicals, being both user and environmentally friendly, thus being considered a “green” technology [10,11]. 

Non-thermal plasma (NTP) processing of seeds can perform low-temperature treatments (which do not thermally affect the integrity and viability of the seeds), influencing all plant development stages. The changes produced could be positive, such as the stimulation of germination, growth, increasing the length of the stems and roots, stimulating the production of antioxidant compounds, or, in some cases, could be negative, inhibiting some or all of the above-mentioned outcomes. Non-thermal plasma acts like a stressor through its charged and uncharged particles, electric field, ultraviolet radiation, reactive species, and so on, among which we believe that at least the reactive species of oxygen and nitrogen (ROS and RON) have a crucial role when the discharge is ignited in atmospheric air. The insights of the mechanisms governing these reported changes are still not fully understood. This research aims to gain some insights about the mechanisms of interaction between non-thermal plasma obtained with different ignition voltages and alfalfa seeds used for sprouting in the case of direct plasma treatment of the seeds. Moreover, based on the existing knowledge, we aim to optimize the plasma processing of the seeds to stimulate plant growth, which is economically important, and the production of nutraceutical compounds, equally if not more important for the consumers targeting foods that feed and heal. 

Sprouts or microgreens have become increasingly popular in people’s diets, especially lately, due to the interest in healthier lifestyles, although they are already popular in many Asian countries and America. Among these, alfalfa (*Medicago sativa*) or lucerne, usually cultivated as a forge crop, gained much attention for its nutraceutical properties. Rich in essential nutrients, alfalfa sprouts are packed with vitamins, minerals, and bioactive compounds that offer numerous health benefits. Some researchers highlighted the significant presence of vitamin K in alfalfa sprouts, which is crucial for bone health and blood clotting mechanisms [12]. Moreover, alfalfa sprouts are abundant sources of vitamin C, a potent antioxidant known for its immune-boosting effects, as well as significant levels of vitamins A, folate, and various B vitamins, contributing to overall health and immune function [13,14,15,16]. These sprouts also contain phytochemicals like saponins and flavonoids, which possess anti-inflammatory and antioxidant properties, as demonstrated by many studies [14,16,17].

Research has further elucidated the potential health-promoting effects of alfalfa sprouts. Studies have demonstrated the cholesterol-lowering properties of alfalfa sprouts, attributed to their high fiber content and saponin compounds, as well as anti-diabetic and anti-obesity properties [17,18,19,20]. Also, alfalfa sprouts contain phytoestrogens, such as coumestrol, which may have protective effects against hormone-related cancers [21]. Incorporating sprouts, such as alfalfa, into the diet offers a convenient way to boost nutritional intake and harness their nutraceutical benefits, making them a valuable addition to a healthy diet. 

Non-thermal plasma treatment applied to different seeds, including alfalfa, has shown significant positive effects on various physiological and biochemical processes, ultimately leading to improved seed performance. Among these, researchers demonstrated the possibility of increasing the germination rate, seedling vigor, and biomass yield of alfalfa plants, enhancing the activities of antioxidant enzymes, such as superoxide dismutase (SOD) and catalase (CAT), in alfalfa seeds, thereby improving their tolerance to abiotic stresses like drought and salinity [22,23,24]. Moreover, NTP treatment has been reported to induce changes in the biochemical composition of sprouts from the *Fabaceae* family, leading to alterations in their nutritional content and phytochemical profiles. For example, our previous study on the NTP treatment of fenugreek showed that in some conditions, NTP treatment of the seeds enhances the production of secondary metabolites, such as flavonoids and phenolic compounds, highlighting the potential of NTP treatment as a sustainable and eco-friendly approach to improving the nutritional value and health-promoting properties of different seeds for the production of sprouts as superfoods with enhanced nutraceutical properties [5]. 

Overall, the literature suggests that NTP treatment holds great promise as a sustainable and effective technology for improving alfalfa sprouts’ growth, quality, and safety, offering potential benefits for both producers and consumers alike. Our previous results show that non-thermal plasma treatment of seeds in different conditions changes the surface of the seeds, influences their germination and the seedlings’ growth, being able to lead in some conditions to an increase of the concentration of nutraceutical compounds in the obtained sprouts. Among a large variety of NTP sources, we chose to focus on a dielectric barrier discharge produced using a flexible electrode, such that the device can also be attached to a package and in-package treatments be performed. Such NTP in a similar configuration proved useful in seed treatments [5,25] and inactivating microorganisms from wrapped medical equipment [6]. Moreover, a simple signal source was used, compared to the usual high-cost, high-voltage devices employed in most existing research. In this work, we focus on alfalfa, scarcely studied despite its aforementioned benefits as a superfood, aiming to reveal the possibility of stimulating the production of nutraceutical compounds in the resulting sprouts. 

## 2. Results

### 2.1. Seed Analyses

#### 2.1.1. Microimaging of the Seed Surface

**Figure 1:**
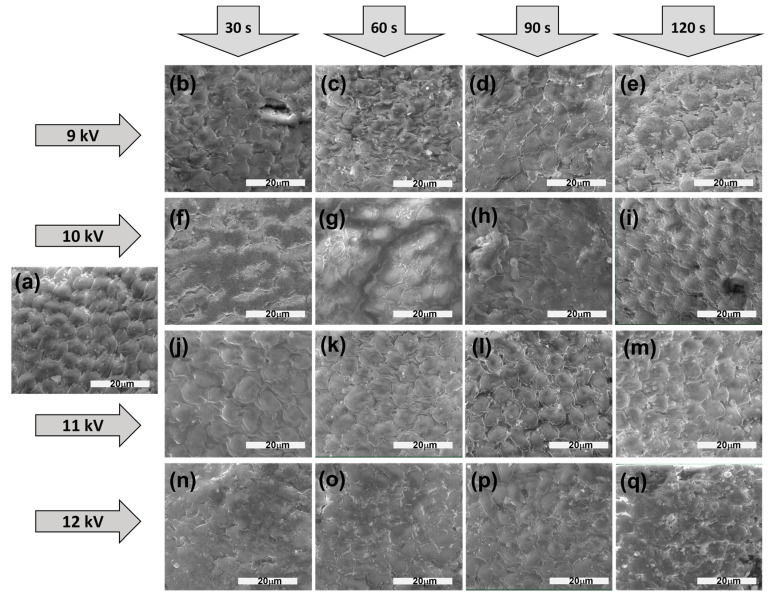
SEM images of the surface of the seeds: (**a**) untreated seeds and treated using 9 kV discharge for (**b**) 30 s, (**c**) 60 s, (**d**) 90 s, (**e**) 120 s, 10 kV discharge for (**f**) 30 s, (**g**) 60 s, (**h**) 90 s, (**i**) 120 s, 11 kV discharge for (**j**) 30 s, (**k**) 60 s, (**l**) 90 s, (**m**) 120 s, and 12 kV discharge for (**n**) 30 s, (**o**) 60 s, (**p**) 90 s, and (**q**) 120 s, respectively.

The surface of alfalfa seeds processed in different conditions changed drastically, with increasing discharge voltage and processing time of the seeds, as seen in Figure 1. NTP produced a strong etching of the surface by removing the seed coat material and producing cracks in the testa. For example, Figure 1f–h,n clearly show a much smoother surface than one of the untreated seeds presented in Figure 1a, while Figure 1c,h,q indicate the presence of big residues removed from the testa.

#### 2.1.2. Water Contact Angle of the Seed Surfaces

**Figure 2:**
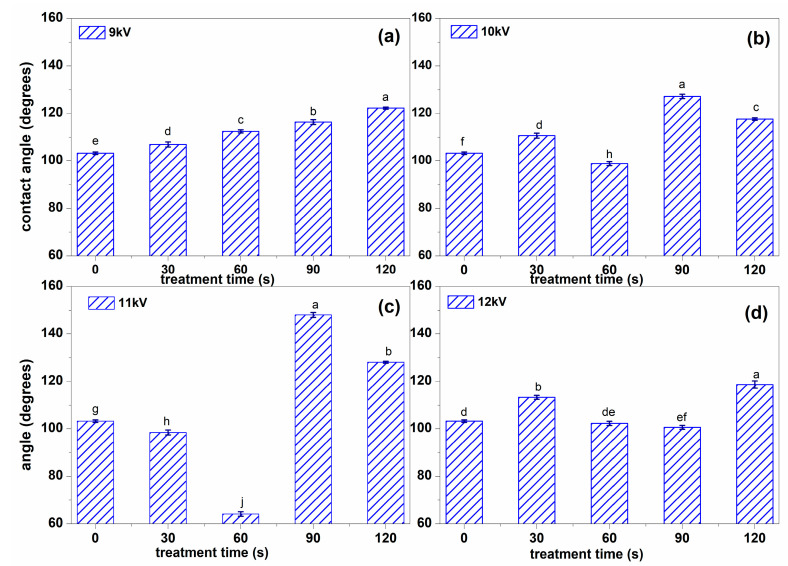
Water contact angle for the surface of the untreated seeds and for those processed in the discharge ignited with (**a**) 9 kV, (**b**) 10 kV, (**c**) 11 kV, and (**d**) 12 kV, respectively, for different processing intervals. Significant differences at 0.05 level are indicated with letters.

To determine how NTP processing influenced the hydrophilicity of the surface of the seeds, the water contact angle measurements were performed using pure water, with results presented in Figure 2. It is clearly seen the difference between the hydrophilicity change in different NTP conditions, with the highest variations for 11 kV ignited NTP that for 60 s led to a decrease of the water contact angle, but for larger processing time in the same voltage conditions led to a considerable increase of the water contact angle, meaning a decrease in the hydrophilicity (Figure 2c). For 12 kV, the changes were the smallest, and there was not a linear behavior as a function of the processing time (Figure 2d) as it happened for the smallest analyzed voltage, 9 kV (Figure 2a). 

### 2.2. Biometric Measurements

**Figure 3:**
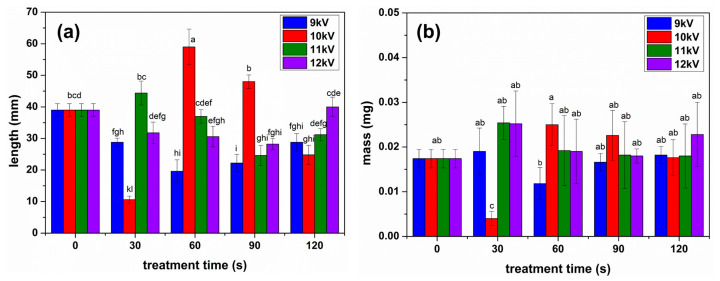
Biometric measurements of the sprouts were obtained after NTP processing of the seeds under different conditions: (**a**) the length of the seedlings and (**b**) the mass of the seedlings. Significant differences at 0.05 level are indicated with letters.

The length of the sprouts obtained after NTP treatment of the seeds is presented in Figure 3a. This indicates that in most conditions, the average length per plant decreased after seed treatment. On the other hand, some conditions proved to be auspicious for sprout growth, especially 60 s for 10 kV NTP, but to some extent also for 30 s in the case of 10 kV NTP. No stimulation was found for 9 kV NTP processing. The average values of the mass of a sprout for different conditions are shown in Figure 3b. Despite the above-mentioned results, when the average length of the sprouts was not higher than the control in most conditions, the mass of the sprouts was just as big or higher than the control in most cases, as presented in Figure 3b. An exceptional situation was recorded in the case of 30 s treatment using 10 kV: the growth of the plants was strongly inhibited, as can be seen from the length and mass measurements. Therefore, it was impossible to determine any biochemical parameters for this condition.

### 2.3. Biochemical Measurements

**Figure 4:**
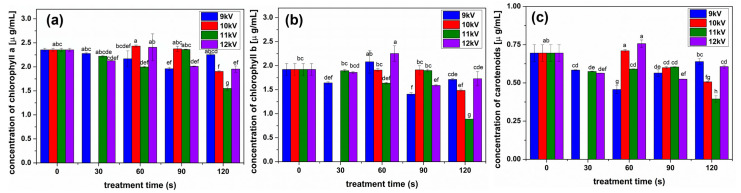
Concentration of (**a**) chlorophyll a, (**b**) chlorophyll b, and (**c**) carotenoids in sprouts obtained from seeds treated with NTP in different conditions compared to untreated seeds. Significant differences at 0.05 level are indicated with letters.

The concentration of chlorophyll pigments was evaluated for the sprouts obtained from seeds processed in different conditions, as shown in Figure 4. Chlorophyll a (Figure 4a) was slightly larger than for control for 9 kV NTP 120 s, 10 kV NTP 60 and 90 s, 11 kV 90 s, and 12 kV 60 s. The concentrations of chlorophyll b in the sprouts significantly increased for 9 kV NTP 60 s and 12 kV 60 s, while for some conditions considerably decreased, such as for 11 kV 60 and 120 s, 9 kV for 30, 90 and 120 s, 10 kV 120 s. In all other conditions, the values were comparable to those of the control. The carotenoid concentrations were inhibited in most cases, except for 10 kV NTP 60 s treatment and 12 kV NTP 60 s treatment. 

**Figure 5:**
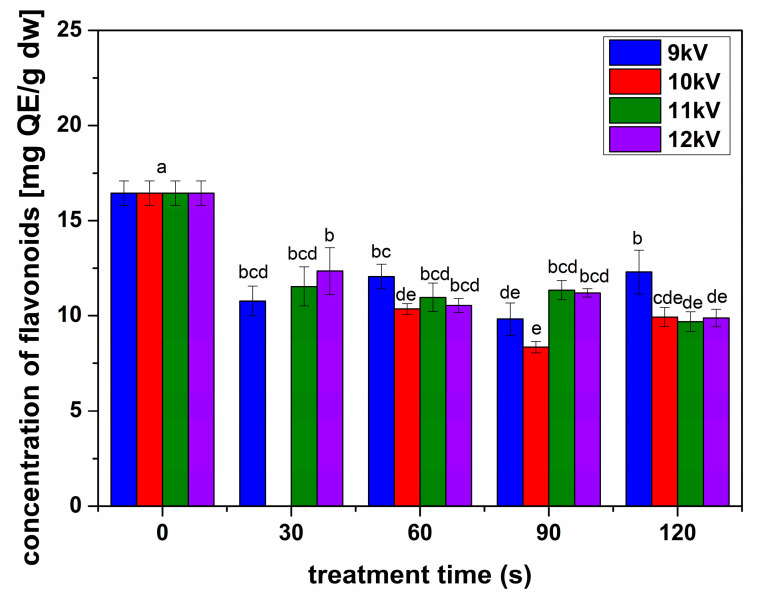
Concentration of flavonoids for the sprouts obtained from seeds treated in different conditions compared with the untreated seeds, as indicated in the figure. Significant differences at 0.05 level are indicated with letters.

**Figure 6:**
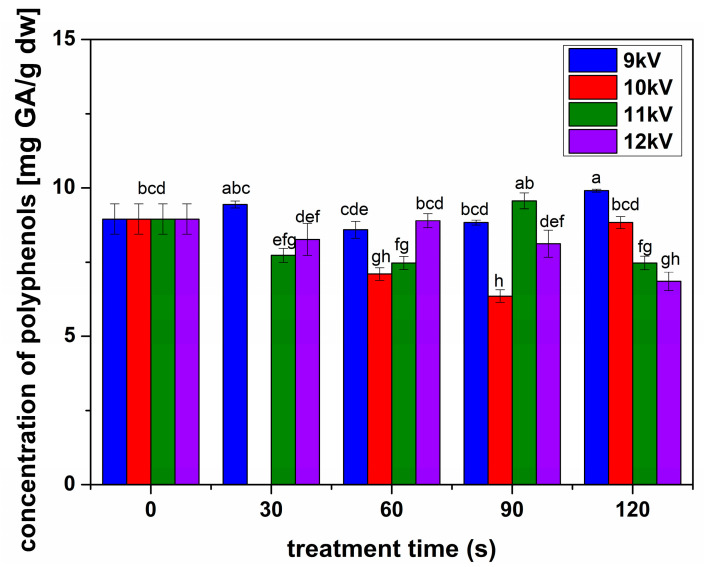
Concentration of polyphenols for the sprouts obtained from seeds treated in different conditions compared with the untreated seeds, as indicated in the figure. Significant differences at 0.05 level are indicated with letters.

**Figure 7:**
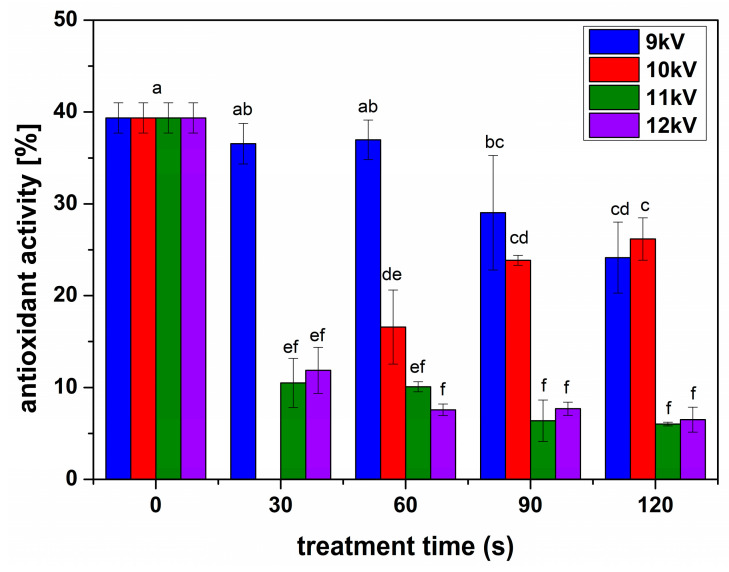
The antioxidant activity of the sprouts obtained from seeds treated in different conditions compared with the untreated seeds, as indicated in the figure. Significant differences at 0.05 level are indicated with letters.

Figure 5 shows the concentrations of flavonoids estimated for all the samples. In all treatment cases, there was a decrease in the concentration of flavonoids. The polyphenol concentrations showed a different behavior than flavonoids, with a slight but significant increase for seeds treated in 9 kV NTP for 30 and 120 s and 11 kV NTP for 90 (Figure 6). The antioxidant activity determined for all samples (Figure 7) indicated an inhibition for all conditions, decreasing with the increase in the processing voltage.

### 2.4. Pearson Correlation

To determine how the NTP processing impacts the studied traits, Pearson correlation was performed for all samples treated in the same NTP conditions for different time intervals. Table 1, Table 2, Table 3 and Table 4 indicate the correlation results for the seed treatments using NTP ignited with 9 kV, 10 kV, 11 kV, and 12 kV HV signals, respectively. For 9 kV, the strongest correlations were between the processing time, water contact angle, time and carotenoids, mass, length, phenols and carotenoids, phenols, and chlorophyll pigment concentration. The correlations are almost similar for 10 kV but with a much stronger correlation between the treatment time, flavonoids, and antioxidant activity. In this case, the treatment time showed strong negative correlations with the chlorophyll pigments. 

## 3. Discussion

NTP treatment of alfalfa seeds clearly changes the microstructure of the seed surfaces, as shown in Figure 1. The changes are not the same for all seed species, in some cases being less obvious; in this case, the material is removed through etching from the surface, which becomes smoother, and even some cracks were evidenced, as in the case of 10 kV NTP processing for 60 s (Figure 1g) or 12 kV 120 s (Figure 1q). Being produced in the air, NTP contains a lot of oxygen and nitrogen reactive species (RONS) that chemically interact with the testa, so do not forget about the physical interaction of NTP species with the surface. We have previously obtained some similar effects in the case of broccoli and fenugreek seeds processed using the same NTP, but other reports are also found in the literature for different NTP discharges and species (such as cotton, rice, wheat) [5,25,26,27,28]. The morphological changes in the testa’s structure definitely depend on the species since this determines the structure of the seed, the thickness of outside layers, and many other traits reflected in the germination and plant growth, as well as the response to NTP treatment. We believe that under NTP treatment of the seeds, the most important is the testa’s structure rather than the seeds’ size, as some of our previous results also showed [25]. The first property influenced by NTP treatment is the hydrophilicity of the seed surface, which will determine its behavior with respect to water. Figure 2 shows that, for a mild treatment when using 9 kV NTP, the water contact angle slightly increases with seed treatment time, results that can be correlated with ESEM imaging. For NTP treatments using higher voltages (Figure 2b,c), the changes are stronger and not linear with respect to the treatment time. 

Sometimes, these changes have been associated with inhibiting germination and plant growth. In the case of alfalfa, the growth of the sprouts obtained from seeds treated for 30 s using 10 kV NTP was strongly inhibited; thus, we can point out the possibility of a connection between the surface morphological changes and the plant growth. Still, for longer processing of the seeds in the same conditions, the growth was stimulated, as seen in Figure 3a, with plants having an average length of about 50% higher than those obtained from untreated seeds. However, we could not find any correspondence between the hydrophilicity of the seeds’ surface and the biometrical traits of the sprouts, indicating that the mechanisms involved in plant growth are more complex to explain than from the increase of hydrophilicity as suggested by some researchers [27]. 

Compared with previous findings of NTP treatments of seeds from other species, alfalfa proved to be mostly inhibited by these, with a significant sprout growth stimulation only in the case of 10 KV NTP treatment, also correlated with the mass of the seedlings (Figure 3b) and an increase of chlorophyll a concentration (Figure 4a) which directly determines the plant growth. NTP can be considered a stress factor, and in our experience with seed treatments, most conditions led to the stimulation of growth correlated with the stimulated production of photosynthetic pigments in most analyzed cases [5,25]. Alfalfa seems to be an exception. Unfortunately, most reported effects of abiotic stress factors are inhibitory when the plants are subjected to different stresses, but there is less insight into what happens when the seeds are subjected to some abiotic stress [29]. On the other hand, pre-treatment of the seeds in some NTPs has shown to be beneficial for the plants, increasing their resistance to different abiotic stress factors mostly due to the RONS that might stimulate the enzyme activity and stimulate the production of bioactive compounds in the plants [29,30,31,32].

Alfalfa NTP treatments were very different compared to previously studied species, with a decrease in flavonoid concentrations in all analyzed conditions (Figure 5). The polyphenols slightly increased in some conditions, especially for long processing with low voltage NTP, while their concentration considerably decreased for higher voltages and longer treatments. We can assume that the threshold processing between stimulation and inhibition was reached very fast in the case of NTP treatments of alfalfa seeds, according to their structure and size, compared to others previously studied, such as broccoli and fenugreek [5,25]. Inhibitory effects have been reported in some NTP treatments, such as rice or wheat [26,33]. It is assumed that the inhibition of the production of antioxidant compounds such as polyphenols and flavonoids, also reflected in the antioxidant activity (Figure 6), is due to the intense oxidative stress induced by the RONS produced, in our case, by NTP [34]. We are inclined to assume that RONS have a crucial contribution when analyzing the antioxidant activity for the analyzed conditions (Figure 7). The antioxidant activity slightly decreased for 9 kV NTP and short processing; then, for longer processing and larger voltages used to produce the NTP, meaning higher concentrations of RONS, the inhibition is much stronger. Of course, RONS are not the only factor determining the outcome; one must also consider that NTP also produces UV, other plasma species, electric fields, etc.

The most interesting discussion that can be made is based on the Pearson correlation results for each NTP condition presented in Table 1, Table 2, Table 3 and Table 4. For 9 kV (Table 1), we see that processing time was very well correlated with the hydrophilicity of the seeds’ surfaces and with carotenoids, mass, phenols, and flavonoids, indicating some small positive responses to NTP treatments. For 10 kV (Table 2), it is very clear that treatment time is strongly negatively correlated to the concentrations of chlorophyll pigments, confirming our previous conclusion that these NTP treatments start to be inhibitory. The conclusion becomes clearer for 11 and 12 kV NTP processing, with strong negative correlations between treatment time and most nutraceutical compounds analyzed, as well as with the length of the sprouts. 

Based on the analyses performed in this work, it is hard to identify the optimum conditions for the stimulation of growth and nutraceutical properties of alfalfa. From the point of view of stimulating the growth, 10 kV 60 s seems the best. If one wants to stimulate the nutraceutical compounds, then 9 kV 30 s could be considered optimum since it does less damage and slightly increases the polyphenols. Direct NTP treatments such as those performed in this work might be too strong for the alfalfa seeds; thus, other approaches could be considered in our future studies, such as an indirect treatment of the seeds. 

## 4. Materials and Methods

### 4.1. Seed Preparation and Sprout Growth

Organic alfalfa seeds were commercially procured (Rapunzel Naturkost GmbH, Legau, Germany). For each condition, an approximate number of 100 seeds were placed on the NTP electrode. Following treatments, the seeds were placed in paper-covered Petri dishes with 2 mL of pure water and incubated at an average temperature of 22 °C (±0.5 °C) and a relative humidity of 15–50% [35]. Some seeds were kept for electron microscopy imaging, and three of each group were used to determine the water contact angle of the testa.

The sprouts were grown for 7 days, and all other analyses were performed after this interval. 

### 4.2. Plasma Device and Treatment Conditions

**Figure 8:**
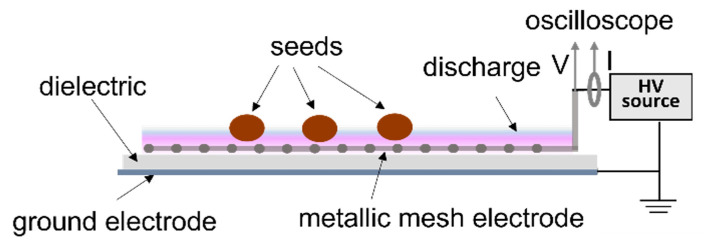
Schematic representation of the NTP device.

For seed treatments, a surface dielectric barrier discharge with a flexible electrode was used [5,25]. A polymeric sheet of 5 mm thickness has one stainless steel 5 × 5 cm electrode on one side and an aluminium tape electrode on the other side (Figure 8); a high voltage signal of 9 up to 18 kV can be applied on these electrodes to produce a non-thermal. The current and voltage were monitored during NTP production using a current probe (Pearson, 4100, Palo Alto, CA, USA) and an HV probe (Tektronix, P6015A, Beaverton, OR, USA), respectively, on an oscilloscope (Rigol DS2072A, Rigol Technologies, Inc., Beijing, China). Seeds were placed on one layer on the grid electrode and processed for 30, 60, 90, and 120 s for NTP ignited in 9, 10, 11, and 12 kV, respectively. 

### 4.3. Seed Analyses

#### 4.3.1. Microimaging and Chemical Analysis of the Seed Surface 

The surface of the seeds untreated and after NTP treatment in all conditions was imaged using environmental scanning electron microscopy (ESEM) performed with a Quanta 450 from FEI (Thermo Fisher Scientific, Hillsboro, OR, USA). The seeds were glued using carbon double tape on the surface of aluminium stubs and then placed in the analysis chamber. The analyses were done in high vacuum conditions (~2.7 × 10^−4^ Pa), using 15 kV electron acceleration voltage at different magnifications. 

#### 4.3.2. Water Contact Angle of the Seed Surface

The water drop method performed the water contact angle of the seeds’ surfaces. A 1 μL pure water drop was placed on the surface of the analyzed seed using a micropipette, and a picture was taken. For each condition, two seeds were analyzed, and the contact angle was determined using imageJ 1.54d software (imageJ.org) with the Drop Snake plugin. In each case, two values (left and right) [36].

### 4.4. Biometric Measurements

After 7 days from NTP treatment, the resulting sprouts were analyzed: the length was measured with a simple ruler, while the mass was measured with a five digits analytical balance (Shimadzu AUW220D, Shimadzu Corporation, Kyoto, Japan).

### 4.5. Biochemical Measurements of Some Nutraceutical Compounds

Determining chlorophyll content in sprouts was done after extracting chlorophyll from 5 g of ground sprout fresh leaves from the same variant, filtered using 96% ethanol, followed by quantification using a spectrophotometer (Specord 210 Plus, Analytikjena, Jena, Germany). After the extraction period, the absorbance of the chlorophyll extracts at 470, 649, and 665 nm were measured against a blank sample. The concentration of chlorophyll a, chlorophyll b, and carotenoids was calculated based on Lichtenthaler formulas adapted by Wellburn [37,38].

The concentration of flavonoids was determined using 0.25 mL of plant extract that was mixed with 5% NaNO_2_ and 10% AlCl_3_ in basic pH (NaOH 1 M), followed by the reading of the absorbance at 510 nm and using a calibration curve made for quercetin (r^2^ = 0.9983) [39].

The concentration of polyphenols was determined after preparing ethanol extracts similar to the extraction of chlorophyll pigments, only for the whole plant to be used. The extracts were sonicated for 15 min, then incubated for 24 h at 4 °C and filtered. The total concentration of polyphenols was evaluated using Folin–Ciocalteu reactive, the concentrations expressed in GA/g dw being calculated from the absorbance at 760 nm, based on a calibration curve obtained for gallic acid (r^2^ = 0.9947) [40].

The antioxidant activity was evaluated after the reduction of 2-diphenyl-1-picrylhydrazyl (DPPH), the reaction with the extract changing the color from violet to yellow; then the absorbance at 510 nm was determined, and results were expressed as a percentage [41].

### 4.6. Statistical Analysis

Significant differences were determined using a one-way variance test (ANOVA) (IBM SPSS v14) for all the measured parameters. When the results were significant (*p* < 0.05), Tukey’s multiple comparison test was used to determine which means from a set are different from the rest. The results are presented as averages with standard deviations; in all cases, letters were used to indicate significant differences determined by Tukey’s test. Pearson correlation test was used to establish eventual correlations between NTP parameters and parameters related to the sprouts. The same software for the statistical analysis was used in this case. 

## 5. Conclusions

In this work, the effects of NTP produced in different voltage conditions were studied with the aim of finding optimum conditions for alfalfa seed treatments. Only a few of the processing conditions proved to be stimulating for the growth and production of some nutraceutical compounds, namely polyphenols and chlorophyll pigments, directly linked to the stimulation of plant growth. We assume that most other inhibitory effects were due to a strong interaction between NTP and the seeds, confirmed by the microimaging of the seeds’ surfaces that were visibly affected by the treatments.

Once again, the results prove that the outcome of NTP treatment also depends on the plant species, with NTP acting as a stress factor. Despite the inhibitory effects, these results are important to get closer to figuring out the insights into the interactions between NTP and seeds as a next step would be figuring out if indirect treatments, which are milder or shorter processing, led to stimulating results in the case of alfalfa. 

## Figures and Tables

**Table 1:** Pearson correlation for 9 kV NTP treatments. Significant positive correlations are denoted in bold.

Correlations 9 kV NTP										
	Time	Contact Ang.	Mass	Length	Phenols	Flavonoids	ch. a	ch. b	Carotenoids	Antiox. Act.
time	1	**0.994** (**)	0.258	−0.100	0.355	0.260	−0.265	−0.219	**0.475**	−0.940
contact ang.	**0.994** (**)	1	0.205	−0.054	0.388	0.363	−0.177	−0.128	**0.460**	−0.921
mass	0.258	0.205	1	**0.527**	**0.675**	−0.474	−0.012	−0.884	**0.909**	−0.569
length	−0.100	−0.054	**0.527**	1	**0.891**	0.294	**0.843**	−0.089	**0.691**	−0.145
phenols	0.355	0.388	**0.675**	0.891	1	0.317	**0.628**	−0.262	**0.899**	−0.578
flavonoids	0.260	0.363	−0.474	0.294	0.317	1	**0.655**	**0.790**	−0.068	−0.100
ch. a	−0.265	−0.177	−0.012	0.843	**0.628**	**0.655**	1	**0.457**	0.244	0.176
ch. b	−0.219	−0.128	−0.884	−0.089	−0.262	**0.790**	**0.457**	1	−0.645	**0.469**
carotenoids	**0.475**	**0.460**	**0.909**	**0.691**	**0.899**	−0.068	0.244	−0.645	1	−0.740
antiox. act.	−0.940	−0.921	−0.569	−0.145	−0.578	−0.100	0.176	**0.469**	−0.740	1

**. Correlation is significant at the 0.01 level (2-tailed).

**Table 2:** Pearson correlation for 10 kV NTP treatments. Significant positive correlations are denoted in bold.

Correlations 10 kV										
	Time	Contact Ang.	Mass	Length	Phenols	Flavonoids	ch. a	ch. b	Carotenoids	Antiox. Act.
time	1	0.648	−0.964	−0.991	**0.682**	−0.204	−0.913	−0.863	−0.999 (*)	**0.958**
contact ang.	**0.648**	1	−0.828	−0.537	−0.115	−0.878	−0.282	−0.173	−0.683	**0.838**
mass	−0.964	−0.828	1	**0.918**	−0.462	**0.458**	**0.771**	**0.696**	**0.975**	−1.000 (*)
length	−0.991	−0.537	**0.918**	1	−0.776	0.068	**0.961**	**0.924**	**0.983**	−0.910
phenols	**0.682**	−0.115	−0.462	−0.776	1	**0.576**	−0.921	−0.958	−0.647	**0.445**
flavonoids	−0.204	−0.878	**0.458**	0.068	0.576	1	−0.212	−0.319	0.250	−0.475
ch. a	−0.913	−0.282	**0.771**	**0.961**	−0.921	−0.212	1	**0.994**	**0.893**	−0.759
ch. b	−0.863	−0.173	**0.696**	**0.924**	−0.958	−0.319	**0.994**	1	**0.838**	−0.682
carotenoids	−0.999 (*)	−0.683	**0.975**	**0.983**	−0.647	0.250	**0.893**	**0.838**	1	−0.971
antiox. act.	**0.958**	**0.838**	−1 (*)	−0.910	**0.445**	−0.475	−0.759	−0.682	−0.971	1

*. Correlation is significant at the 0.05 level (2-tailed).

**Table 3:** Pearson correlation for 11 kV NTP treatments. Significant positive correlations are denoted in bold.

Correlations 11 kV										
	Time	Contact ang.	Mass	Length	Phenols	Flavonoids	ch. a	ch. b	Carotenoids	Antiox. Act.
time	1	**0.604**	−0.727	−0.724	0.170	−0.802	−0.600	−0.745	−0.700	−0.933
contact ang.	**0.604**	1	−0.233	−0.667	**0.701**	−0.162	0.089	−0.119	−0.268	−0.847
mass	−0.727	−0.233	1	**0.851**	−0.336	**0.408**	0.223	0.316	0.122	**0.623**
length	−0.724	−0.667	**0.851**	1	−0.740	0.189	−0.083	0.092	0.020	**0.814**
phenols	0.170	**0.701**	−0.336	−0.740	1	**0.447**	**0.684**	**0.518**	**0.465**	−0.463
flavonoids	−0.802	−0.162	**0.408**	0.189	**0.447**	1	**0.959** (*)	**0.995** (**)	**0.942**	**0.579**
ch. a	−0.600	0.089	0.223	−0.083	**0.684**	**0.959** (*)	1	**0.977** (*)	**0.919**	0.325
ch. b	−0.745	−0.119	0.316	0.092	**0.518**	**0.995** (**)	**0.977** (*)	1	**0.961** (*)	**0.517**
carotenoids	−0.700	−0.268	0.122	0.020	**0.465**	**0.942**	**0.919**	**0.961** (*)	1	**0.544**
antiox. act.	−0.933	−0.847	**0.623**	**0.814**	−0.463	**0.579**	0.325	**0.517**	**0.544**	1

*. Correlation is significant at the 0.05 level (2-tailed). **. Correlation is significant at the 0.01 level (2-tailed).

**Table 4:** Pearson correlation for 12 kV NTP treatments. Significant positive correlations are denoted in bold.

Correlations 12 kV										
	Time	Contact ang.	Mass	Length	Phenols	Flavonoids	ch. a	ch. b	Carotenoids	Antiox. Act.
time	1	0.195	−0.438	**0.550**	−0.755	−0.827	−0.583	−0.364	0.166	−0.872
contact ang.	0.195	1	**0.756**	**0.895**	−0.751	−0.110	−0.490	−0.391	0.355	0.154
mass	−0.438	**0.756**	1	0.387	−0.258	**0.557**	−0.266	−0.355	−0.039	**0.762**
length	**0.550**	**0.895**	0.387	1	−0.856	−0.542	−0.462	−0.260	**0.571**	−0.297
phenols	−0.755	−0.751	−0.258	−0.856	1	**0.465**	**0.838**	**0.673**	−0.117	0.373
flavonoids	−0.827	−0.110	**0.557**	−0.542	**0.465**	1	0.061	−0.205	−0.640	**0.941**
ch. a	−0.583	−0.490	−0.266	−0.462	**0.838**	0.061	1	**0.962** (*)	**0.443**	0.122
ch. b	−0.364	−0.391	−0.355	−0.260	**0.673**	−0.205	**0.962** (*)	1	**0.643**	−0.107
carotenoids	0.166	0.355	−0.039	**0.571**	−0.117	−0.640	0.443	0.643	1	−0.372
antiox. act.	−0.872	0.154	**0.762**	−0.297	0.373	**0.941**	0.122	−0.107	−0.372	1

*. Correlation is significant at the 0.05 level (2-tailed).

## Data Availability

The data presented in this study are available on request from the corresponding author.
